# Intravitreal ranibizumab injection combined trabeculectomy versus Ahmed valve surgery in the treatment of neovascular glaucoma: assessment of efficacy and complications

**DOI:** 10.1186/s12886-016-0248-7

**Published:** 2016-05-26

**Authors:** Lan Liu, Yongfeng Xu, Zhu Huang, Xiaoyu Wang

**Affiliations:** Department of Ophthalmology, the First Affiliated Hospital, College of Medicine, Zhejiang University, 79# Qingchun Road, Hangzhou, People’s Republic of China; Department of Neurology, the Second Affiliated Hospital, School of Medicine, Zhejiang University, Hangzhou, People’s Republic of China

**Keywords:** Neovascular glaucoma (NVG), Ranibizumab, Vascular endothelial growth factor (VEGF), Trabeculectomy, Ahmed valve surgery

## Abstract

**Background:**

Researches have shown anti-vascular endothelial growth factor (VEGF) agent is effective in treating neovascular eye diseases. The purpose of this study is to evaluate the efficacy and safety of intravitreal ranibizumab (IVR) injection combined trabeculectomy in the treatment of neovascular glaucoma (NVG), and compared it with Ahmed valve surgery.

**Methods:**

Thirty-six NVG patients (37 eyes) from the First Affiliated Hospital of Zhejiang medical college, between January 1, 2014 and January 31, 2015, were included in this prospective, interventional clinical study. Eighteen NVG eyes were given IVR injection one week before trabeculectomy. Ahmed valve implantation surgery was performed in nineteen eyes. Ocular pain, best corrected visual acuity (BCVA), intraocular pressure (IOP) and surgical complications were evaluated before and after the surgery.

**Results:**

IOP was significantly decreased following IVR injection combined trabeculectomy treatment (baseline 57.1 ± 8.9 mmHg; week 1, 15.2 ± 4.3 mmHg *p* = 0.000; month 1, 16.9 ± 2.1 mmHg *p* = 0.000; month 3, 20.3 ± 7.7 mmHg *p* = 0.000; month 6, 19.7 ± 7.3 mmHg *p* = 0.000). There was a significant, though modest, BCVA improvement in sighted eyes of IVR group (baseline 2.42 ± 0.68, W1 1.80 ± 0.91, *P* = 0.013; M1 1.77 ± 0.93, *p* = 0.011). IVR injection combined trabeculectomy had less postoperative complications and lower failure ratio than Ahmed surgery (IVR 5.6 %, Ahmed 31.6 %).

**Conclusions:**

The study revealed that IVR injection combined trabeculectomy was an effective and safe treatment for NVG. Compared with Ahmed surgery, IVR injection combined trabeculectomy had less complications and higher success ratio. (Chinese Clinical Registry, TRN ChiCTR-OPN-16008147, 3/24/2016, retrospectively registered)

## Background

Neovascular glaucoma (NVG) is an intractable glaucoma secondary to the neovascularization of the iris and the anterior chamber angle. It is caused by ischemia and hypoxia of the retina in ocular ischemic diseases like proliferative diabetic retinopathy (PDR), central retinal vein occlusion (CRVO), etc. NVG is a serious ocular disorder with poor prognosis [1, 2]. The management of NVG is still difficult. Conventional treatments including filtering surgery, cyclophotocoagulation, cryotherapy, aqueous drainage device, panretinal photocoagulation may still have a high risk of failure to control the intraocular pressure (IOP) and prevent deterioration of the disease [1–3].

Vascular endothelial growth factor (VEGF), a protein induced by hypoxia and ischemia, is an important regulator in angiogenesis and inflammation responses [4]. VEGF was found highly expressed in neovascular membranes and ocular fluids of neovascular ocular diseases such as PDR, CRVO and also NVG [5, 6]. Therefore, VEGF inhibitors were effective in treating neovascular eye diseases. Ranibizumab (Lucentis), the Fab fragment of recombinant humanized IgG1kmurine monoclonal antibody against VEGF-A, was approved for the treatment of ocular neovascular diseases such as wet age-related macular degeneration [7, 8]. The utilization of ranivizumab has been expanded to treat many diseases with macular edema such as PDR, CRVO and NVG in recent years [9, 10]. There are several studies about bevacizumab, another anti-VEGF agent off-label used in ocular neovascular diseases, which has served as an effective medicine in treating NVG [11–13]. But clinical trials about the efficacy and safety of the ranibizumab in treating NVG are relatively rare.

Our study was designed to test the efficacy of intravitreal ranibizumab (IVR) injection combined with trabeculectomy with mitomycin C (MMC) on patients with NVG. We also compared its efficacy and safety with the conventional treatment, Ahmed valve surgery, to evaluate advantages and disadvantages of these two therapies separately.

## Methods

### Patients and inclusion criteria

The study was a prospective interventional study including 36 patients with 37 NVG eyes. Randomized placebo-controlled design was not conducted because the poor prognosis of NVG left no room for ethical randomization. NVG patients were treated in the ophthalmology department of the First Affiliated Hospital of Zhejiang medical college, Zhejiang University, Hangzhou, China, between January 1, 2014 and January 31, 2015. The study was approved by the Ethics Committee of the First Affiliated Hospital of Zhejiang University and it was performed in accordance with the tenets of the Declaration of Helsinki. All patients were aware of their therapy. An informed consent form was signed by every patient.

Patients were included in the study based on the following criteria: (i) age > 20 years; (ii) diagnosed as NVG with rubeosis; (iii) IOP > 21 mmHg with the maximum use of anti-glaucoma drugs. Exclusion criteria included: (i) active ocular infection; (ii) previous glaucoma filtering surgery; (iii) previous anti-VEGF treatment; (iv) any contraindication of intraocular injection or surgery, such as high risk of bleeding, pregnancy, and infection, et al.

NVG patients received one of two types of treatment: IVR injection and subsequent trabeculectomy with MMC, or Ahmed valve implantation surgery. Main medical history and previous treatments were recorded.

### IVR injection and surgical techniques

All surgeries and intravitreal injections were performed by Dr. Xiaoyu Wang and Dr. Lan Liu. Eighteen eyes with NVG received IVR injection under topical anesthesia in the aseptic condition of an operating room. 0.5 mg (0.05 ml) Ranibizumab was injected through the pars plana with a 25G needle. The patients were given topical antibiotics and previous antiglaucoma medicines for 1 week after injection. The surgery of trabeculectomy with MMC was performed 1 week later. The globe was pulled inferiorly by a traction suture. The conjunctival incision was made along the limbus to create a fornix-based conjunctival flap in the superotemporal quadrant. A half thick 4 mm × 4 mm square scleral flap was made. MMC (0.4 mg/ml) soaked sponge was placed under the scleral flap for 1 to 2 min. Then the area was irrigated with plenty of saline. Trabecular meshwork (1 × 1.5 mm) was cut and the peripheral iridectomy (1 × 1 mm) was preformed. The scleral flap was closed with two 10-0 nylon sutures at its corners. The conjunctiva was sutured with 8-0 vicryl sutures.

Ahmed valve implantation surgery was performed on nineteen NVG eyes and the procedure was described as follows. A fornix-based conjunctival and Tenon’s flap was created in the superotemperal quadrant. The Ahmed valve implantation (Model FP7, New World Medical, Inc.) was inserted into the Tenon’s capsule and fixed on the sclera 8 mm posterior to the limbus with 8-0 sutures. A 4 mm × 4 mm square scleral flap was made, a corneoscleral track was made by a 23-gauge needle and the tube was inserted into the anterior chamber through the scleral flap. The tube was fixed to the episclera with 8-0 sutures. Topical eyedrops of antibiotics and steroids were used in every patient for two weeks.

### Outcome measurement and follow-up

Pain of the patients was assessed and recorded by numerical rating scale (NRS), ranging from 0 (no pain) to 10 (worst imaginable pain). Best corrected visual acuity (BCVA), IOP (Goldmann applanation tonometer), angle status and full ophthalmic examination were taken before and after the surgery. BCVA was recorded using logMAR equivalent, and counting fingers was assigned value 2, hand movement was assigned 3. The new vessels of iris were observed by slip lamp. Surgical complications and postoperative anti-glaucoma medications were also recorded. The patients were followed up for at least 6 month and the IOP and BCVA were recorded on day 1, week 1, then monthly after the operation. The staff members performing the NRS, BCVA and IOP assessment were not involved in implementing the surgeries.

The efficacy of the treatment was evaluated by the success of the surgery. Complete success was defined as IOP ≥ 6 mmHg and ≤ 21 mmHg without any anti-glaucoma medications or further glaucoma surgery, and without loss of light perception. Partial success was defined as IOP < 21 mmHg with topical anti-glaucoma medicines. Surgical failure was defined as IOP ≥ 21 mmHg even with anti-glaucoma medicines, or additional surgical treatment was needed to control IOP, or loss of light perception.

### Statistical analysis

Statistical analysis was conducted using the software SPSS 10.0 (SPSS, Inc. Chicago, IL). Independent and paired Student t-test was used to assess differences between groups. Results were presented as mean ± standard deviation (SD). *P* < 0.05 was considered statistically significant.

## Results

### Baseline characteristics

Thirty-six NVG patients (37 eyes) were included in the study. Eighteen NVG eyes underwent IVR injection and subsequent trabeculectomy (IVR group). Nineteen eyes underwent Ahmed valve implantation surgery (Ahmed group). The patients’ demographics and basic characteristics were summarized in Table 1. There were no differences in the gender and age between the two groups. Of all 37 NVG eyes, 14 (37.8 %) were caused by PDR, 18 (48.6 %) by CRVO, 1 (2.7 %) by branch retinal vein occlusion (BRVO), 1 (2.7 %) by ischemic optic neuropathy, and 3 (8.1 %) were after the intraocular surgery. There were no differences among the causes between two groups. The baseline IOP was 57.1 ± 8.9 mmHg in the IVR group and 49.8 ± 11.8 mmHg in the Ahmed group. Baseline BCVA of sighted eyes was 2.42 ± 0.68 in the IVR group and 2.51 ± 0.84 in the Ahmed group. No differences were found in baseline IOP (*p* = 0.12) and BCVA (*p* = 0.68) between the two groups. Sixteen eyes (88.9 %) in the IVR group and 17 eyes (89.5 %) in the Ahmed group were given retinal photocoagulation before the operation, and no differences were found between the two groups. Two eyes in the IVR group and 3 eyes in the Ahmed group were given additional retinal photocoagulation after the operation to prevent deterioration due to the diseases. There were 3 patients (1 in the IVR group, 2 in the Ahmed group) who experienced enucleation in month 3 due to the uncontrolled high IOP and unbearable eye pain, and were lost to follow-up.

**Table 1:** Baseline characteristics of included patients

	IVR and trabeculectomy with MMC (*n* = 18)	Ahmed valve surgery (*n* = 19)	*P* value
Age (y)	62.3 ± 10.8	56.7 ± 13.6	0.54
Sex			
Male Female	11(61.1 %) 7(38.9 %)	10(52.6 %) 9(47.4 %)	0.74
Systemic diseases			
Hypertention Diabetes mellitus	11(61.1 %) 6(33.3 %)	12(63.2 %) 9(47.4 %)	
Causes of NVG			
PDR CRVO	6(33.3 %) 10(55.6 %)	8(42.1 %) 8(42.1 %)	
BRVO		1(5.3 %)	
After intraocular surgery	2(11.1 %)	1(5.3 %)	
Ischemic optic neuropathy		1(5.3 %)	
Lens status			
Phakic Intraocular lens	16(88.8 %) 2(11.1 %)	13(68.4 %) 6(31.6 %)	
Baseline IOP (mmHg)	57.1 ± 8.88	49.8 ± 11.8	0.12
BCVA (logMAR)			
NLP LP others	3 2 2.42 ± 0.68	3 3 2.51 ± 0.84	0.68
Pain grade (NRS)	6.44 ± 0.78	5.68 ± 1.00	0.33

Abbreviations: *NVG* neovascular glaucoma, *PDR* proliferative diabetic retinopathy, *CRVO* central retinal vein occlusion, *BRVO* branch retinal vein occlusion, *IOP* intraocular pressure, *BCVA* best corrected visual acuity, *NLP* no light perception, *LP* light perception, *IVR* intravitreal ranibizumab, *MMC* mitomycin C

### Rubeosis regression and pain relief

**Fig. 1:**
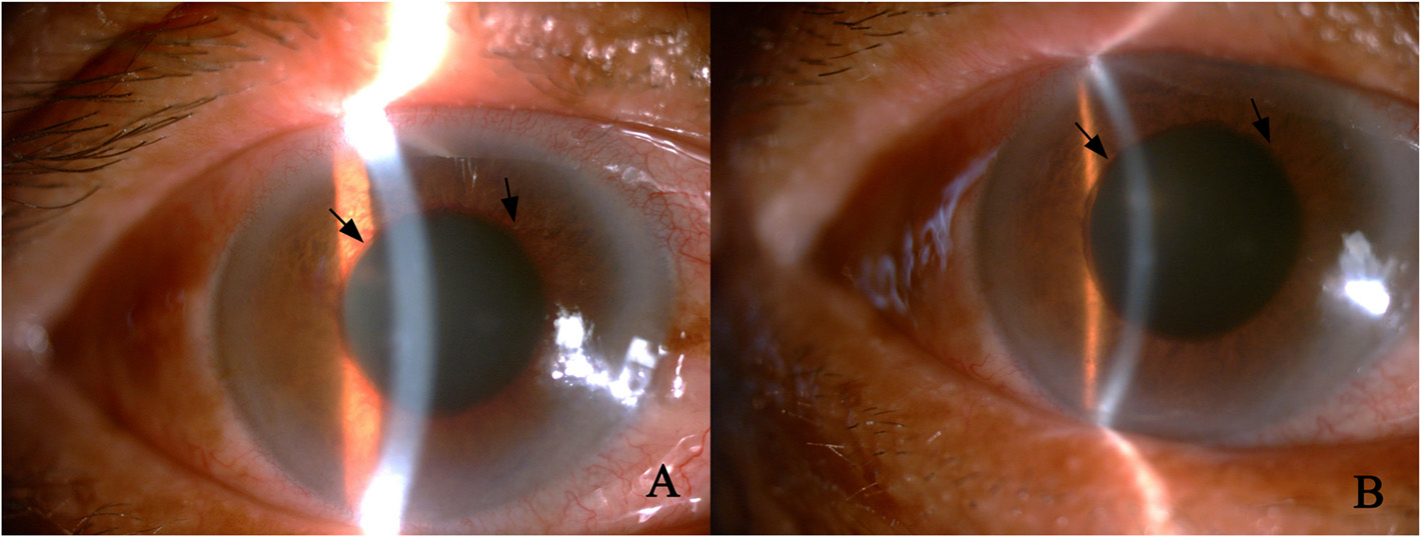
Anterior segment photography of a neovascular glaucoma patient before and after intravitreal ranibizumab injection (IVR). **a**. Massive new vessels (black arrows) were seen on the iris around the pupil before IVR. **b**. New vessels were regressed (black arrows) three days after IVR from the same patient of A

Rubeosis was found in all 37 NVG eyes (100 %) and hyphema in 3 patients (8.1 %) before treatment. In the IVR group, the new vessels of the iris apparently regressed in all 18 eyes after the IVR injection (Fig. 1). The eye pain (NRS score) was significantly relieved one week after trabeculectomy (before surgery 6.4 ± 0.8; one week 2.2 ± 0.5 *p* = 0.000), and had almost disappeared one month later(0.3 ± 0.5, *p* = 0.000). In the Ahmed group, the pain was also significantly decreased at one week (5.7 ± 1.0; one week, 2.6 ± 0.96, *p* = 0.000) and almost vanished one month later (0.5 ± 0.7, *p* = 0.000, Table 2). The NRS score remained low in both groups at month 3 and month 6, except 1 patient in IVR group, 2 in Ahmed group had increased NRS score (IVR 7, Ahmed 8) owing to the uncontrolled IOP.

**Table 2:** NRS scores and BCVA of neovascular glaucoma patients

	IVR group (*n* = 18)	Ahmed group (*n* = 19)
NRS scores		
Before surgery 1 week after surgery 1 month after surgery	6.4 ± 0.8 2.2 ± 0.5 0.3 ± 0.5	5.7 ± 1.0 2.6 ± 0.96 0.5 ± 0.7
BCVA (sighted eyes)		
Before surgery 1 week after surgery 1 month after surgery	2.42 ± 0.68 1.80 ± 0.91 1.77 ± 0.93	2.51 ± 0.84 1.92 ± 1.24 2.53 ± 0.85

Abbreviations: *IVR* intravitreal ranibizumab, *NRS* numerical rating scale, *BCVA* best corrected visual acuity

### IOP

**Fig. 2:**
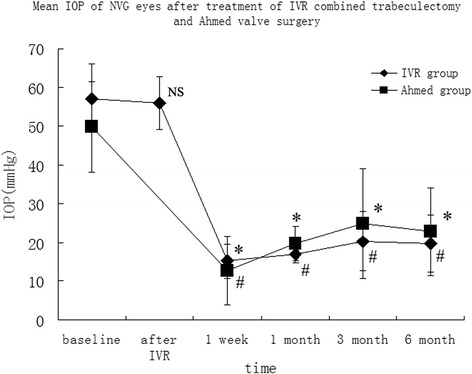
Mean intraocular pressure (IOP) before and after the surgery in intravitreal ranibizumab injection (IVR) group and Ahmed surgery (Ahmed) group. *, *P* < 0.05 compared with the baseline of IVR group, #, *P* < 0.05 compared with the baseline of Ahmed group. NS, *P* > 0.05 compared with the baseline of IVR group

In the IVR group, IOP was significantly decreased (week 1, 15.2 ± 4.3 mmHg *p* = 0.000; month 1, 16.9 ± 2.1 mmHg *p* = 0.000; month 3, 20.3 ± 7.7 mmHg *p* = 0.000; month 6, 19.7 ± 7.3 mmHg *p* = 0.000) after trabeculectomy compared with the baseline IOP (57.1 ± 8.9 mmHg). But IOP was only slightly lowered after IVR injection (55.9 ± 6.9 mmHg, *p* = 0.154). There was also a significant drop of IOP in the NVG eyes of the Ahmed group (W1 12.8 ± 8.7 mmHg *p* = 0.000, M1 19.7 ± 4.5 mmHg *p* = 0.000, M3 24.9 ± 14.2 mmHg *p* = 0.000, M6 22.8 ± 11.2 mmHg *p* = 0.000) (Fig. 2.). Results showed that IOP was significantly lower in the IVR group than that in the Ahmed group at month 1 (*P* = 0.021). There were no differences of IOP between the two groups at week 1 (*P* = 0.302), month 3 (*P* = 0.225) and month 6 (*P* = 0.324).

### BCVA

Visual acuity was relatively low in all NVG patients. Mean BCVA of sighted eyes (*n* = 26) was 2.46 ± 0.75 (IVR 2.42 ± 0.68, Ahmed 2.51 ± 0.84), and 5 eyes showed light perception (LP), 6 had no light perception (NLP) (Table 1). Results showed that there was a significant BCVA improvement at week 1 and month 1 in the IVR group (W1 1.80 ± 0.91, *P* = 0.013; M1 1.77 ± 0.93, *p* = 0.011) compared with the baseline, though the improvement was relatively modest. BCVA was not significantly increased in the Ahmed group (W1 1.92 ± 1.24, *P* = 0.156; M1, 2.53 ± 0.85, *p* = 0.252, Table 2). Visual acuity of all NVG patients in both groups maintained stable after month 1, except 1 patient in the IVR group and 2 in the Ahmed group who lost light perception due to the uncontrolled IOP at month 3.

### Complications

There were no intraoperative complications in either the IVR or the Ahmed group. There were apparently more postoperative complications in the Ahmed group than in the IVR groups (Table 3), especially in the early postoperative period (within two weeks of the surgery). Six eyes were found with low IOP (hypotony, IOP ≤ 5 mmHg), 2 eyes with shallow anterior chamber, 1 eye with no anterior chamber which needed additional surgery, and 1 eye with hyphema and 1 eye with exudative inflammation at week 1 in the Ahmed group. There were no early complications in the IVR group. In the late postoperative period (after two weeks), 3 eyes (16.7 %) with complications (hyphema, vitreous hemorrhage and high IOP) were found in the IVR group. There were obviously more complications in the Ahmed group (8 eyes, 42.1 %).

**Table 3:** Intraoperative and postoperative complications

	IVR group (*n* = 18)	Ahmed group (*n* = 19)
intraoperative	0	0
Early postoperative^a^		
Shallow or no anterior chamber hypotony Hyphema Exudative inflammation	0 0 0 0	3(15.8 %) 6(31.6 %) 1(5.3 %) 1(5.3 %)
subtotal	0	11(57.9 %)
Late postoperative^b^		
Hyphema Vitreous hemorrhage endophthalmitis Shallow or no anterior chamber High IOP Tube occlusion by iris	1(5.5 %) 1(5.5 %) 0 0 1(5.5 %) 0	0 2(10.5 %) 0 1(5.3 %) 3(15.8 %) 2(10.5 %)
subtotal	3(16.7 %)	8(42.1 %)
total	3	19

^a^Early postoperative: within two weeks after the surgery; ^b^Late postoperative: after two weeks of the surgery

In the IVR group, 11 eyes (61.1 %) were maintained IOP < 21 mmHg without anti-glaucoma medications (complete success), 6 eyes (33.3 %) maintained IOP < 21 mmHg with medications (partial success), 1 eye (5.6 %) failed to control IOP even with anti-glaucoma medications. In the Ahmed group 11 eyes (57.9 %) had complete success, 2 eyes (10.5 %) partial success and 6 eyes (31.6 %) failed.

## Discussion

NVG is a serious and refractory glaucoma with poor prognosis. There are no optimal treatments that would cure the disease. Conventional treatment would still fail due to the recurrence of neovascularization [14]. In our study, we evaluated the efficacy of IVR injection combined with trabeculectomy in treating NVG patients, and which we also compared with treatment of Ahmed valve surgery. Our results showed that IVR injection evidently reduced the new vessels of the iris, IVR combined trabeculectomy effectively relieved the ocular pain, controlled IOP, and partially improved BCVA. Compared with the Ahmed valve surgery, the IVR combined trabeculectomy treatment had less postoperative complications and lower failure ratios.

NVG is caused by the neovascularization and fibration of the anterior chamber angle and iris, and results in uncontrolled IOP and ocular pain. Proper management should include the treatment of the underlying disease and the high IOP [2, 15]. Trabeculectomy was considered the most effective surgical procedure for reducing IOP in patients with primary open-angle glaucoma and primary angle-close glaucoma [16, 17]. But the management of NVG is still highly challenging and controversial. Trabeculectomy alone had a high risk of failure to control IOP of NVG, due to the bleb adhesion by neovascularization in the anterior chamber angle [2]. Improper treatment would finally result in blindness and intractable ocular pain. In managing NVG, it is important to treat underlying causes in addition to the elevated IOP [15]. VEGF is the key angiogenic factor in the pathogenesis of neovascular ocular diseases such as NVG [18]. Inhibition of VEGF also markedly reduced the fibroblast proliferation and scar formation after glaucoma filtration surgery [19, 20]. Therefore, anti-VEGF antibody served as a useful adjunctive to the therapy of NVG via its anti-angiogenic and anti-fibrotic properties [21]. In our study, we treated 18 NVG eyes with anti-VEGF drugs (ranibizumab) before the trabeculectomy with MMC. We found that IVR injection could significantly decrease the iris neovascularization and partly lower IOP. Since neovascularization was the main cause of NVG, IVR injection might provide chances to increase the success probability of the subsequent anti-glaucoma surgery.

Recent studies have shown outstanding efficacy of anti-VEGF agents (bevacizumab, ranibizumab) in treating neovascular eye diseases, such as age-related macular degeneration [7, 8]. Several studies have shown that bevacizumab injection was a useful adjuvant for the treatment of NVG [11, 12]. Intraocular injection of bevacizumab could significantly reduce the new vessels in the anterior chamber angle, lower IOP and decrease the aqueous VEGF concentration [22, 23]. The study by Klettner and Roider revealed ranibizumab was more efficient than bevacizumab in neutralizing VEGF in vitro [24]. And due to the off-label use of bevacizumab in eye diseases, ranibizumab (Lucentis) should be more suitable in treating NVG. There were no randomized controlled trials (RCTs) using anti-VEGF agents for the treatment of NVG [25], while there are a few studies about uses of ranibizumab in NVG. Artilces by Elmekawey and Kitnarong [26, 27] showed that intracameral or intravitreous injection of ranibizumab combined trabeculectomy effectively controlled IOP of NVG. A study by Li et al. [28] found that IVR combined vitrectomy, lensectomy, retinal photocoagulatin and trabeculectomy could control IOP and improve BCVA for NVG patients with vitreous hemorrhage. Luke et al [29] used IVR alone and showed, with repeated injections, it was beneficial for treating NVG. Desai et al [30] found that intravitreal injection of ranibizumab was an effective adjunctive treatment to Ahmed tube surgery in open-angle glaucoma. In our study, we conducted intravitreal injection of ranibizumab before trabeculectomy, and compared its efficacy and complications with the conventional Ahmed valve surgery. Our results revealed that the treatment of IVR injection combined trabeculectomy induced apparent regression of iris new vessels, significantly relieved eye pain, lowered IOP and partially improved BCVA in NVG patients. The high ratio of complete and partial success supported the efficacy of IVR injection combined trabeculectomy in treatment of NVG.

A Glaucoma drainage device (such as Ahmed valve implantation) was usually chosen as a common option to treat the secondary glaucoma in complex cases like NVG [2]. We compared the efficacy and complications of IVR + trabeculectomy treatment with the treatment of Ahmed valve surgery on NVG in our study. Our results showed that while Ahmed valve surgery also significantly relieved the eye pain, lowered IOP and partially improved BCVA, compared with IVR injection with trabeculectomy, Ahmed valve surgery markedly increased the postoperative complications, especially in the early postoperative stage, such as shallow or no anterior chamber, hyphema and hypotony. These complications ultimately increased the failure probabilities of the Ahmed surgery. Results showed that there was lower failure ratio in the IVR group compared with the Ahmed group.

Overall our study showed that IVR combined trabeculectomy would be an effective and safe treatment for NVG. Further clinical trials with larger sample numbers are needed to provide more evidence to define this optimal treatment of NVG.

## Conclusions

Our study demonstrated IVR injection combined trabeculectomy was an effective and safe treatment for NVG. Compared with Ahmed surgery, IVR injection combined trabeculectomy had less complications and higher success ratio.

## Abbreviations

BCVA, Best corrected visual acuity; BRVO, branch retinal vein occlusion; CRVO, central retinal vein occlusion; IOP, intraocular pressure; IVR, intravitreal ranibizumab; MMC, mitomycin C; NRS, numerical rating scale; NVG, Neovascular glaucoma; PDR, proliferative diabetic retinopathy; RCTs, randomized controlled trials; VEGF, Vascular endothelial growth factor.
